# Chrononutrition against Oxidative Stress in Aging

**DOI:** 10.1155/2013/729804

**Published:** 2013-06-18

**Authors:** M. Garrido, M. P. Terrón, A. B. Rodríguez

**Affiliations:** Department of Physiology (Neuroimmunophysiology and Chrononutrition Research Group), Faculty of Science, University of Extremadura, 06006 Badajoz, Spain

## Abstract

Free radicals and oxidative stress have been recognized as important factors in the biology of aging and in many age-associated degenerative diseases. Antioxidant systems deteriorate during aging. It is, thus, considered that one way to reduce the rate of aging and the risk of chronic disease is to avoid the formation of free radicals and reduce oxidative stress by strengthening antioxidant defences. Phytochemicals present in fruits, vegetables, grains, and other foodstuffs have been linked to reducing the risk of major oxidative stress-induced diseases. Some dietary components of foods possess biological activities which influence circadian rhythms in humans. Chrononutrition studies have shown that not only the content of food, but also the time of ingestion contributes to the natural functioning of the circadian system. Dietary interventions with antioxidant-enriched foods taking into account the principles of chrononutrition are of particular interest for the elderly since they may help amplify the already powerful benefits of phytochemicals as natural instruments with which to prevent or delay the onset of common age-related diseases.

## 1. Introduction

Understanding the aging process has gained in importance with people's increasing life expectancy. Chronological age is the strongest predictor of chronic diseases, and the scientific community is searching for protective agents that can contribute to preventing or delaying the onset of many age-related diseases.

The complexity of the holistic systems of which living cells form a part makes it difficult to distinguish between the causes and consequences of aging. Indeed, it is still unknown whether this process derives from a single or multiple causes [1]. Theories of aging are mainly divided into those assuming that aging is genetically encoded and those assuming that it is due to a decline in maintenance mechanisms and exponential accumulation of molecular damage resulting in degeneration and dysfunction at the cellular level [2]. Among the latter theories, the free radical theory of aging (also known as oxidative stress theory) put forward by Harman in 1956 [3] has received extensive support. It posits that the organism's deterioration resulting from increasing longevity is above all a consequence of the persistent accumulation of free radical mediated damage to essential molecules. This accumulation gradually compromises cell and tissue function, and eventually the entire function of the organism itself [4]. Within this theory, some authors argue that aging results from damage caused by free radicals to nuclear DNA, while others argue that it is a result of alterations to, and progressive loss of, mitochondria as a result of the mutilation of their DNA, thus reducing their biological effectiveness [1]. Indeed, mitochondrial DNA lacks polyamines or protective histones and, thus, is more susceptible than nuclear DNA to oxidative damage. Mutations are, therefore, more likely to take place in the mitochondrial genome of differentiated cells [5]. Curiously, healthy elderly individuals can have oxidative stress levels that are similar to those of young adults [6], or at least comparable in terms of antioxidant defences [7]. This suggests that oxidation is not inevitable in aging.

It is commonly argued that aging is not a genetically controlled process but an interaction between environment and genes [8]. Psychological stress and lifestyle factors appear to have an impact on the level of oxidation [9, 10]. Stress has been the most extensively studied negative factor in the brain's vulnerability to aging. In contrast, positive environmental factors such as a healthy diet can lead to improvements in aging [11]. Indeed, it is estimated that a third of all cancer deaths in the United States could be avoided through appropriate dietary modification [12]. Changes in dietary behaviour, such as increased consumption of fruits, vegetables, and grains, are a practical strategy for significantly reducing the incidence of chronic diseases.

Phytochemicals are bioactive nonnutrient compounds present in fruits, vegetables, grains, and other plant foods. They have been linked to reductions in the risk of major oxidative stress-induced diseases [13]. Numerous investigations have shown a strong link between dietary intake of phytochemicals and reduced risk of cancer and cardiovascular disease worldwide. Thus, a prospective study in Finland involving 9959 men and women (ages 15–99 years) found an inverse association between the intake of flavonoids and the incidence of cancer [14]. After a 24-year follow-up, the risk of lung cancer was reduced by 50% in the highest quartile of flavonol intake. As well as flavonoids, other phenylpropanoids, isoprenoids, and indoleamines, particularly the indole melatonin, merit particular attention due to their biological activities [15].

Melatonin is the principal neurohormone secreted at night by the vertebrate pineal gland. It is an important component of the body's internal timekeeping system [16]. In particular, it is a signal of darkness that encodes time of day and length of day information for the brain [17]. A conceptual difficulty in melatonin research is that, while it is a signal of darkness, it has different functional consequences depending on the given species' time of peak activity. In nocturnal species, it is associated with arousal and physical activity. In diurnal species, it is associated with sleep and rest [16].

**Figure 1:**
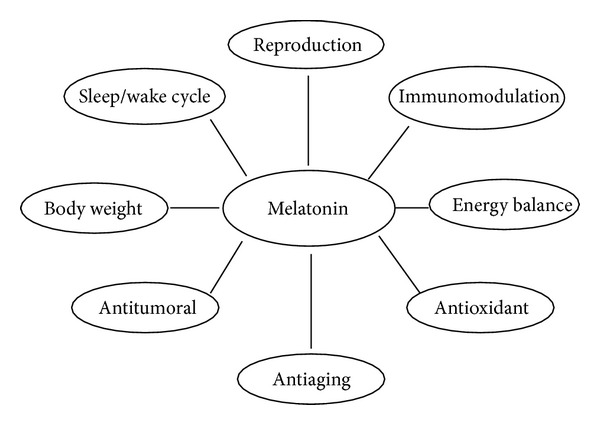
Physiological functions modulated by the indole melatonin.

In diurnal animals, the onset of melatonin secretion is closely associated with the timing of sleep propensity. It also coincides with decreases in core body temperature, alertness, and performance [18]. For this reason, it is believed to play a part in sleep initiation as the trigger for opening the circadian “sleep gate,” thus acting as a sleep regulator [19]. In this respect, the efficacy of melatonin supplementation to combat sleep disorders is well known, especially in the elderly with their marked reduction in melatonin production [16]. This hormone has a broad spectrum of physiological effects [20]. These include, but are not limited to, chronobiological, immunomodulatory, neuroendocrine, and antioxidant activities (Figure 1). All of these may contribute to the observed anti-aging potency of this natural agent [21].

Related to its antioxidant activities, melatonin acts as a potent antioxidant and free radical scavenger [22–25]. It not only scavenges the especially toxic hydroxyl radicals, but also performs indirect antioxidant actions via its ability to stimulate antioxidative enzymes [26]. Melatonin diminishes free radical formation at the mitochondrial level by reducing the leakage of electrons from the electron transport chain [27]. Increasing the levels of circulating melatonin, either directly by exogenous administration or indirectly by including vegetables rich in this compound in the diet, enhances the individual's antioxidant status [28–31]. It also stimulates a number of antioxidative enzymes which metabolize reactive products to innocuous agents. As well as for diseases in which there is an elevated production of free radicals, this may have implications for aging since production of this pineal indole wanes with increasing age. Indeed, some authors speculate that its loss contributes to the aging process [4].

Some components of foods possess biological activities which influence circadian rhythms in humans. But also “when” the food is consumed influences the normal functioning of biological rhythms. A central target of current chronobiological research is how nutrients can alleviate or even prevent diseases. In the present review, we shall focus on the potential use of chrononutrition as a novel dietary strategy to counteract the deleterious actions of free radicals and reactive species on physiological systems during aging.

## 2. Chronobiology: At the Cutting Edge of Health Sciences

We humans are immersed in an environment characterized by repetitive rhythmic cycles [32]. Conditions that are modified by different temporal cycles include organic efficiency, pathologies and the pharmacokinetics, pharmacodynamics, and efficacy of drugs. These cycles also entrain various processes, both physiological (body temperature, hormone and neurotransmitter secretion, and immune, cardiovascular, and digestive functions) and behavioural (sleep/wake cycle, activity/rest periods, digestion and excretion, sensory perception, learning, and memory), to a well-defined rhythm [33]. This phenomenon of rhythmicity extends to all classes of living beings, whether animals or plants, and at all levels of organization (molecules, cells, tissues, and organs).

Chronobiology is the discipline that studies the nature and function of biological rhythms, defined as the recurrence of any event within a biological system at roughly regular intervals [34]. The human body's biological clocks, for example, are controlled by synchronization with signals from the external environment [35].

The entire spectrum of biological rhythms covers an extremely wide range of frequencies [36]. All organisms, however, present circadian rhythms. These are biological processes that have an endogenous, entrainable oscillation of about 24 hours. They affect most of the organism's physiological functions—the sleep/wake cycle, immune function, melatonin secretion, and the production and secretion of numerous neurotransmitters [37, 38]. The internal synchronization provided by circadian clocks may be altered by many factors, one of which is aging. The aging process leads to a situation of chronobiological imbalance that results in shortening the period and reducing the amplitude of the oscillator, loss of the circadian rhythm itself, appearance of an ultradian pattern, and internal desynchronization [39, 40]. During aging, any disturbance or imbalance in the relationship between the circadian and homeostatic systems may lead to the impairment of numerous physiological processes.

Antioxidant systems also deteriorate during aging. Elderly individuals, therefore, become more vulnerable to pathological conditions related to oxidative stress and may require an extra supply of dietary antioxidants to combat free radicals [41]. Previous studies by our research group and by other workers have shown that neuroendocrine and immune disorders due to the aging process may be ameliorated by supplementation with melatonin, serotonin, and/or their precursor, the amino acid tryptophan [42–46]. These are all compounds with powerful antioxidant properties [23, 29, 47]. The administration of tryptophan increases the availability of serotonin in the brain [46, 48]. Melatonin levels are consequently elevated as well [49]. Besides being an antioxidant, melatonin also has oncostatic, immunomodulatory, anti-inflammatory, and chronobiotic properties [50–52]. Given this context, the administration of tryptophan and melatonin in accordance with the needs of elderly subjects may contribute to readjusting any disturbances they have in their circadian rhythms.

**Figure 2:**
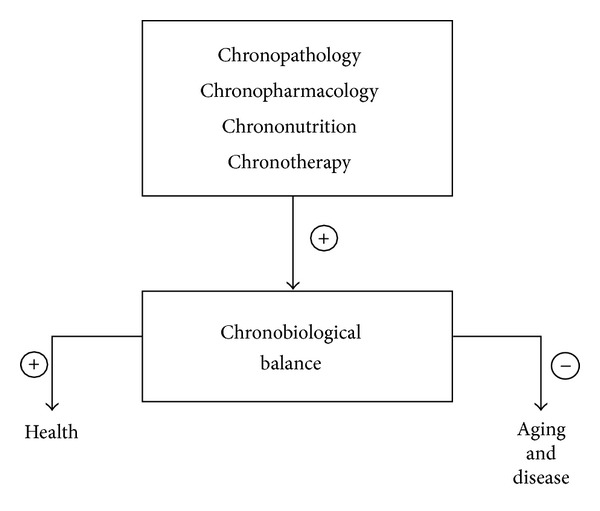
Influence of chronobiology applications on aging and disease.

Knowledge of the nature and function of biological rhythms is of practical as well as theoretical interest. This is reflected in the growing number of applications of chronobiology published in the recent health sciences literature. One such novel area of research is chronopharmacology. This is focused on the design and evaluation of drug delivery systems that release a bioactive agent at a rhythm matching the biological requirements of the treatment of a given disease [53]. Applying the knowledge of circadian function and regulation to the relevance of disease has enabled a chronobiology-based approach in the timing of administration of conventional drugs in order to synchronize the rhythms in disease activity with the efficacy of a particular drug, thus allowing for its optimal efficacy in the patient. Other recent applications of chronobiology include chronopathology, chronotherapy, and chrononutrition, all aimed at reducing the need for invasive methods in therapeutic interventions, and therefore are of unquestionable importance (Figure 2).

## 3. Basis of Chrononutrition: Health Benefits

Our eating schedules are dictated not only by food supply, hunger, and satiety, but also by convenience and social habits and pressures. Feeding behaviour is the first element to consider in an organism's nutritional process. The vast majority of studies have focused on examining the homeostatic regulation of the quantity and quality of food ingested. The hypothalamus is the main neural structure involved. It acts in close correlation with the release of such hormones as cholecystokinin (CCK), leptin, ghrelin, and insulin. Temporal aspects of this regulation have been far less studied [54]. Meal timing can affect many physiological processes. The sleep/wake cycle, core body temperature, performance, alertness, and secretion of many hormones are examples. Since these are functions with a circadian rhythmicity, they deteriorate during aging with the weakening of overt circadian patterns [20]. That meal timing has major effects on the body has led to the conviction that, in choosing food, it is not only convenient to consider its nutritional value, but also its capacity to promote or hinder the normal functioning of the circadian cycle's control systems (Table 1). In humans, alterations have been detected in the overall expression of daily rhythms when food intake is limited to the usual period of rest (i.e., at night), as occurs during Ramadan [54].

Nutrients and phytochemicals play an essential part in the regulation of such circadian functions as sleep. Compared with a control diet high in carbohydrates and low in fat, a very low carbohydrate, fat-rich diet has been found to reduce the proportion of rapid eye movement (REM) sleep recorded by polysomnography. But it also increased the percentage of deep slow-wave (NREM) sleep [55]. While the total amount of carbohydrates may influence the architecture of sleep, it does not affect the duration. However, the evidence on whether carbohydrates positively impact sleep quality is not completely consistent, since consuming carbohydrate meals with high or low glycaemic loads seems not to affect any polysomnographically determined sleep index [55]. On the contrary, some components of the typical human diet, such as vitamin B12, improve alertness and concentration and reduce the daytime sleepiness phase [56]. Since some nutrients can entrain the circadian rhythm, diet design must take meal timing into account as well as the quantity and quality of the foods. In particular, the time of day at which food is consumed directly influences certain metabolic and hormonal factors—glucose, free fatty acids, glucocorticoids, and thyroid hormones, among others [57]. These ideas can be subsumed under the concept of chrononutrition. This concept reflects that it is not only the content of food, but also the time of ingestion and the interactions of its nutritional components which naturally contribute to the proper functioning of the circadian system.

### 3.1. Clinical Applications of Chrononutrition in Oxidative Stress

Harman's free radical theory of aging posits that oxidized macromolecules accumulate with age, resulting in decreased function and shortened life span [3]. Indeed, reduction of oxidative stress has been found to be associated with prolongation of life expectancy in many organisms [58–60]. Avoiding the formation of free radicals and reducing oxidative stress, thereby strengthening the body's antioxidant defences, can reduce the rate of aging and the risk of age-associated diseases [61]. In this sense, the primary prevention of chronic diseases through dietary modification may be just as effective as the secondary treatments that are commonly employed and less costly [62].

The possibility that mammalian life span could be significantly extended by diet modification was first demonstrated in a rodent study published by McCay and coworkers in 1935. This seminal experiment showed that life span can be extended by diet restriction without malnutrition, as opposed to diet restriction with malnutrition which can have the opposite effect [63]. Not only diet restriction, but also dietary patterns can have important long-term benefits for health. The abundance of bioactive phytochemical compounds provided by the fruits, vegetables, wine, and olive oil that make up the typical Mediterranean dietary pattern has proven to be effective in ameliorating some aging conditions associated with oxidative stress [64]. The tocopherols, carotenoids, and vitamin C present in pigmented and citrus fruits, and in such vegetables as carrots, tomatoes, broccoli, and red peppers, are positively correlated with a lower incidence of coronary heart disease [65]. Olive oil contains ubiquinol and tocotrienols which inhibit low-density lipoprotein (LDL) oxidation and reduce the risk of cancer [66]. Other dietary components, such as vitamin E or specific forms of fatty acids, especially (*n* − 3) polyunsaturated fatty acids (PUFAs), contribute to the modulation of the immune and inflammatory systems, helping to prevent infectious and inflammatory diseases in the elderly [67]. Finally, in 1997 the most significant phenol in red wine, trans-resveratrol, was shown to prevent carcinogenesis in mice [68]. Since then, this phytochemical has been shown to have various pharmacological properties, namely, antioxidative [69], anti-inflammatory [70], anti-diabetic [71], anti-asthmatic [72], and antalgic [73], observed both *in vitro* and *in vivo* [74]. The doses applied in human trials examining specific health benefits of resveratrol range from 5 mg to 5 g, and some have considered additional compounds with putative synergistic effects [75]. A major critical limitation of most of the clinical research on resveratrol has been the lack of trials examining the longer-term health effects of this compound. The examples we have briefly discussed above are just a few of the more than several thousands of bioactive compounds that have been identified in foodstuffs from all over the world [76].

The vast majority of studies on the biological functions of phytochemicals have focused on analysing their antioxidant and/or anti-inflammatory properties. Few of them have taken into account the chrononutritional properties responsible for the effectiveness of such compounds. In humans, the type of food (macronutrient) is a temporally controlled variable. Once the nutrient enters the bloodstream, it may resemble the behaviour of a drug. It is thus subject to the principles of chronopharmacology, the most important of which is that the time of day influences both beneficial and unwanted effects. All the processes involved in nutrition display circadian and ultradian patterns which include rhythms of food intake, gut motility, secretion of digestive juices, absorption of digested foods, production of key metabolic enzymes, and energy expenditure. Consequently, different nutrients tend to be absorbed in different proportions, depending on the time of day [54].

Given their potent antioxidant activity, the tryptophan, serotonin, and/or melatonin content of foodstuffs and foodstuff-type beverages such as almonds, nuts, sweet cherries, apples, corn, beer, and olive oil [77–82] may have major implications for animal and human health. This is particularly the case in combating a variety of disorders and diseases in which there is an elevated production of free radicals [83–85]. The antioxidant effects of these compounds have been demonstrated both in animal models and in humans. In animals, a Jerte Valley cherry-based beverage, rich in tryptophan, serotonin, and melatonin, has proven to be an efficient antioxidant-enriched product. Delgado et al. [86] showed that consumption of this product augmented the circulating levels of both melatonin and serotonin. This augmentation was positively correlated with increases in serum antioxidant capacity in both rats (*Rattus norvegicus*, a nocturnal animal) and ringdove (*Streptopelia risoria*, a diurnal animal) and in both young and old age groups. Its consumption also modulated the balance of pro- and anti-inflammatory cytokines, especially in the old animals, by downregulating the levels of proinflammatory cytokines and upregulating those of anti-inflammatory cytokines [87]. The clear importance of this type of finding is that nutritional interventions could delay or even prevent the functional deterioration of the immune system that accompanies aging [88]. The therapeutic properties shown by this cherry-based beverage may be attributed to the high melatonin content of the Jerte Valley cherry, although the involvement of other antioxidants, such as polyphenols, cannot be ruled out.

**Figure 3:**
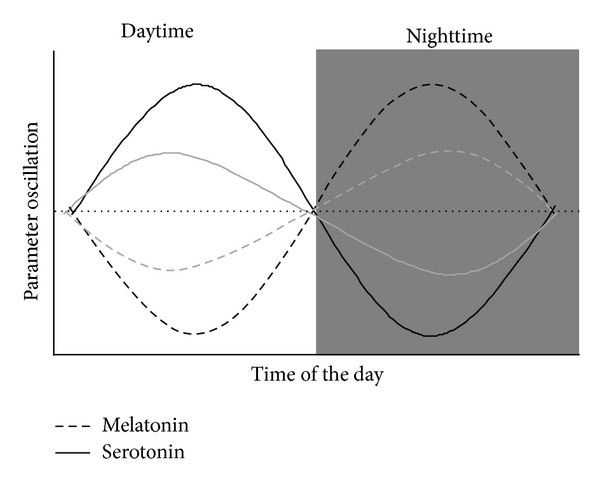
Circadian rhythms of melatonin and serotonin. Black lines represent normal circadian patterns of melatonin and serotonin secretion, while grey lines show a disturbed pattern due to aging process.

Given these results with animal models, tests were conducted with humans. It was found that both a diet enriched with Jerte Valley cherries and the ingestion of a Jerte Valley cherry-based nutraceutical product improve the antioxidant status of young, middle-aged, and elderly individuals [31, 89]. Particularly noteworthy was that not only the substantial amount of melatonin, serotonin [78], and tryptophan [80] contained in these Jerte Valley cherries, but also the timing of the meal, were critical to achieving the beneficial effects of these dietary interventions. Since serotonin and melatonin have opposite circadian rhythms, for example, serotonin levels peak during the day while those of melatonin peak at night (Figure 3), the cherries and the cherry-based product were consumed twice a day, once at lunch and once as supper desserts. The lunchtime consumption of cherries or the cherry-based product was designed both to directly increase the diurnal circulating serotonin concentration and to indirectly increase the nocturnal circulating melatonin concentration by enhancing the amount of serotonin available to be converted into melatonin at night [31, 90]. Hence, together with the ingestion of cherries (or the cherry-based product) at supper time, this boosts the total nighttime circulating melatonin levels [31, 91]. These interventions succeeded in improving the subjects' antioxidant status. The likelihood that this was indeed due to increases in the melatonin and serotonin concentrations was confirmed indirectly by determining the urine 6-sulfatoxymelatonin (aMT6-s) and 5-hydroxyindoleacetic acid (5-HIAA) levels. Most importantly, both of these nutritional interventions showed sleep-promoting and mood-enhancing actions in the young, middle-aged, and elderly subjects, which correlated with the increments in melatonin and serotonin levels, respectively [31, 90, 91].

### 3.2. Clinical Implications of Chrononutrition in Aging

Aging is a complex process. It is related to circadian rhythm disruption with the resulting sleep disturbances [92] and other physiological and psychological dysfunctions [93]. These include impaired nutrient absorption [94, 95], immunosenescence [96–98], decreased hormone levels [99], and neuronal death [100, 101]. The age-associated disruption in the sleep/wake rhythm has been linked to the conventional decline in melatonin levels in advancing age (Figure 3). It may be corrected by supplementation with melatonin or with foodstuffs rich in melatonin (or in melatonin's precursors). In this respect, Bravo et al. [102] have shown that the consumption of tryptophan-enriched cereals increases aMT6-s and 5-HIAA levels, with the consequent improved mood and sleep quality in elderly subjects. Given the mood-enhancing and wakefulness-promoting properties of serotonin during the daytime, and the sleep-regulating functions of tryptophan, serotonin, and melatonin at night, these cereals were consumed twice a day (at breakfast and supper).

There is clear evidence that aging is associated with elevated basal morning levels of circulating glucocorticoids, such as cortisol [103]. It is commonly believed that altered HPA activity,  for example, increased glucocorticoid activity or decreased brain serotonin concentration, is associated with several age-related pathologies [104]. In this sense, the consumption of the aforementioned Jerte Valley cherry product was effective in diminishing cortisol levels in young, middle-aged, and elderly subjects, the decline being especially more pronounced with advancing age. Increases in plasma tryptophan availability have been shown to enhance positive mood and dampen the cortisol response after an acute exposure to experimental stress. The mechanism is via enhancement of the brain serotonin functions that are involved in the adaptation to stress [105, 106]. Consequently, the tryptophan and serotonin present in the Jerte Valley cherry product may contribute to reducing cortisol levels, particularly in the elderly who are more vulnerable to stress.

It is noteworthy that many studies of the health-promoting effects of the antioxidant-rich fruits and foodstuff-type beverages present in the Mediterranean diet report better outcomes in advanced ages. Thus, González-Flores et al. [107, 108] find that the consumption of both plums (*Prunus salicina* Lindl. cv. Crimson Globe) and grape juice elevated the aMT6-s levels and the antioxidant capacity determined from the urine in young, middle-aged, and elderly individuals, with the effects being greater with advancing age. Likewise, Garrido et al. [109] reported that the inclusion of lycopene (the most potent *in vitro* antioxidant of the carotenoids) in a virgin olive oil enhanced the antioxidant effects of its ingestion. Again, the increases in this antioxidant capacity were positively correlated with age. The explanation may be that while energy requirements decrease with advancing age, nutritional requirements increase because of the greater demand for maintenance of the functionality of physiological systems [110].

## 4. Concluding Remarks

Just as research has provided a solid foundation for the overall relationship between food and health, the links between specific food components and particular health risks are now also being confirmed. This has had a direct impact on the food processing industry which has begun to turn its attention to the development of nutraceutical products. The marketing of the first infant milk formula that took into account the circadian variations of the different components present in breast milk clearly contributed to bringing science and industry together. This artificial infant milk (Blemil Plus^®^) is a dissociated formula consisting of different nutritional components that promote wakefulness, such as vitamins A, C, E, and B12 (Blemil 1 Plus day; N° WO2006/034955; PCT/EP2005/054516), and others that help to improve the effectiveness and quality of sleep, such as tryptophan, medium-chain triglycerides, and the nucleotides uridine and adenosine, among other constituents (Blemil 1 Plus night; N° WO2006/034955; PCT/EP2005/054516). A ground-breaking clinical study in the field of nutrition was the demonstration that the implementation of these chronobiologically adapted formulas in the diet indeed contributes to consolidating the sleep/wake cycle in the newborn [111].

In general, dietary interventions with antioxidant-enriched foods based on the principles of chrononutrition are particularly relevant for the elderly, since this population commonly experiences progressive deterioration in physiological functions and metabolic processes. The aforementioned tryptophan-enriched cereals and the Jerte Valley cherry product have been shown to have especially beneficial effects on sleep quality and antioxidant status in the elderly. These nutritional strategies may contribute to taking full advantage of the potential benefits of phytochemicals as natural instruments with which to prevent or delay the onset of common age-related diseases.

Most clinical studies conducted in the field of chrononutrition have so far focused their attention on small groups of the population with no marked health problems. Clearly therefore, larger-scale studies are required on priority groups of the population, for example, cancer or obesity patients, to examine the clinical relevance of the supplementation of diets with chronobiotically enriched foodstuffs. Such studies would also help adapt the elaboration of diets and foodstuffs to the needs of specific populations according to age, sex, health goals, lifestyle, and genetic predisposition to certain diseases.

## Figures and Tables

**Table 1:** Examples of antioxidant requirements according to chrononutrition principles.

	Which?	Where?	When?
Antioxidants	Vitamins (C and E)	Kiwi (*Actinidia chinensis*); broccoli (*Brassica oleracea*)	Daytime
	Carotenoids (lycopene)	Watermelon (*Citrullus lanatus*); tomato (*Lycopersicon esculentum*)	Daytime
	Phytoalexin (resveratrol)	Grape (*Vitis vinífera*); almond (*Prunus amygdalus*)	Daytime
	Tryptophan	Apple (*Malus domestica*); lentil (*Lens culinaris*)	Daytime/nighttime
	Serotonin	Banana (*Musa sapientum*); coffee (*Coffea arabica*)	Daytime/nighttime
	Melatonin	Cherry (*Prunus avium*); nut (*Juglans regia*)	Nighttime
